# Effectiveness of Electrostatic Shielding in High-Frequency Electromagnetic Induction Soil Sensing

**DOI:** 10.3390/s22083000

**Published:** 2022-04-14

**Authors:** Dorijan Špikić, Matija Švraka, Darko Vasić

**Affiliations:** Faculty of Electrical Engineering and Computing, University of Zagreb, 10000 Zagreb, Croatia; matija.svraka@fer.hr

**Keywords:** electromagnetic induction, soil sensing, high-frequency, electrostatic shielding, electrical conductivity

## Abstract

High-frequency electromagnetic induction (HFEMI) sensors, operating in the frequency range from 300 kHz to 30 MHz, have been proposed for the measurement of soil electrical conductivity and dielectric permittivity that are related to the physical and chemical properties of soil. Because of the high-frequency operation, the capacitive coupling between the sensor transmitter and receiver coils is comparable to inductive coupling, creating the need for electrostatic shielding. The remnant capacitive coupling after the implementation of shielding can lead to significant difficulties in the sensor signal interpretation, because both coupling mechanisms are highly dependent on the geometry of the HFEMI sensor and applied shield. In this paper, we introduce the discussion on the relationship between the sensor geometry, shielding and the coupling mechanisms for HFEMI soil sensing. We theoretically and experimentally evaluate a typical HFEMI sensor and its shielding in the frequency range of up to 20 MHz and propose a method for evaluating the effectiveness of a shield configuration. In the case study, we experimentally analyze the HFEMI sensor above a saline solution for two shield configurations. The results agree well with the results of a finite element method analysis.

## 1. Introduction

Electromagnetic induction (EMI) sensing is used as a noncontact method for the measurement of soil electromagnetic parameters (electrical conductivity, dielectric permittivity and magnetic susceptibility) related to physical and chemical properties such as soil salinity, water content and density [1,2,3]. Most EMI soil sensors consist of transmitter and receiver coils at the intercoil separation corresponding to the soil exploration depth [4,5]. They operate in the low-frequency range below 300 kHz, where they are sensitive to electrical conductivity and magnetic susceptibility [6]. In contrast to low-frequency EMI, high-frequency electromagnetic induction (HFEMI) sensors operate in the frequency range between 300 kHz and 30 MHz [7]. They exhibit a higher sensitivity to electrical conductivity and dielectric permittivity, translating them into richer information on soil properties [8,9,10]. The application of HFEMI to soil sensing was proposed in [7]. The HFEMI soil sensor system presented in [11] used an excitation frequency of 1.56 MHz and geometry resembling the commercially available low-frequency EMI soil sensors [12]. The sensitivity and lift-off analysis of the HFEMI sensor in the frequency range of up to 10 MHz was presented in [13]. Apart from soil sensing, the HFEMI method has also been used to detect buried objects with a low-metal content or low conductivity (<100 kS/m) [14] and for the spectroscopic or tomographic inspection of biological tissue with a conductivity of less than 10 S/m [15,16].

The induced voltage in a receiver coil is a superposition of two components: a primary voltage resulting from the direct coupling between the transmitter and receiver, and a secondary voltage due to the coupling with the induced fields in the soil. In addition to this inductive coupling mechanism, there is also a capacitive coupling caused by parasitic capacitances between the transmitter, receiver and the inspected medium [10]. A common issue with HFEMI sensors is that capacitive coupling becomes comparable to inductive coupling as the excitation frequency increases. Capacitive coupling affects both the primary and secondary voltage, complicating the sensor data interpretation [8].

Electrostatic shielding is a standard procedure for minimizing capacitive coupling [17,18]. Most EMI sensing studies, unrelated to soil sensing, approach this problem from the practical design perspective, and little further attention is given to its effectiveness or the comparison of various shielding techniques and materials. For example, in the tomography system of [19], transmitter and receiver coils were wrapped with an audio cable, and in [20], copper tape was used as an electrostatic shield. The tomography system in [21] was developed with a patterned shield etched on a printed circuit board (PCB) to minimize the capacitive coupling between the PCB coils and the tested medium. In the spectroscopy system developed in [22], a conductive graphite paint was applied to the walls between the coils and the material under test to serve as a low conductivity electrostatic shield. To the best of our knowledge, there are no studies on HFEMI soil sensing that address the capacitive coupling and the effectiveness of electrostatic shielding.

Inductive and capacitive coupling mechanisms are highly dependent on the geometry of the HFEMI sensor and the shielding used, and it can be difficult to distinguish their contributions to the sensor response [11]. Following the analysis in [10], in this paper, we evaluate electrostatic shielding theoretically and experimentally on a typical sensor configuration for EMI soil sensing in the frequency range from 1 MHz to 20 MHz. Based on this, we propose a method for evaluating the effectiveness of a shield configuration. We demonstrate this method on several PCB shield designs. As a case study, we compare the laboratory measurement results for two shielding configurations with a finite element method (FEM) study for the case where the sensor was positioned above the saline solution with an electrical conductivity of 1 S/m and a dielectric permittivity of 78. The significance of this study is that it introduces the discussion on the relationship between sensor geometry, shielding and the coupling mechanisms in HFEMI soil sensing. The contributions of the paper are the model of coupling mechanisms for the typical HFEMI soil sensor, the method for evaluating shielding effectiveness and its experimental and FEM validation.

The paper is organized as follows: In Section 2, we describe the experimental setup, its equivalent circuit model and the method for estimating the model parameters. In Section 3, we present and discuss the estimated receiver and transmitter circuit parameters, the estimated coupling parameters and the case study results.

## 2. Materials and Methods

### 2.1. Experimental Setup

**Figure 1:**
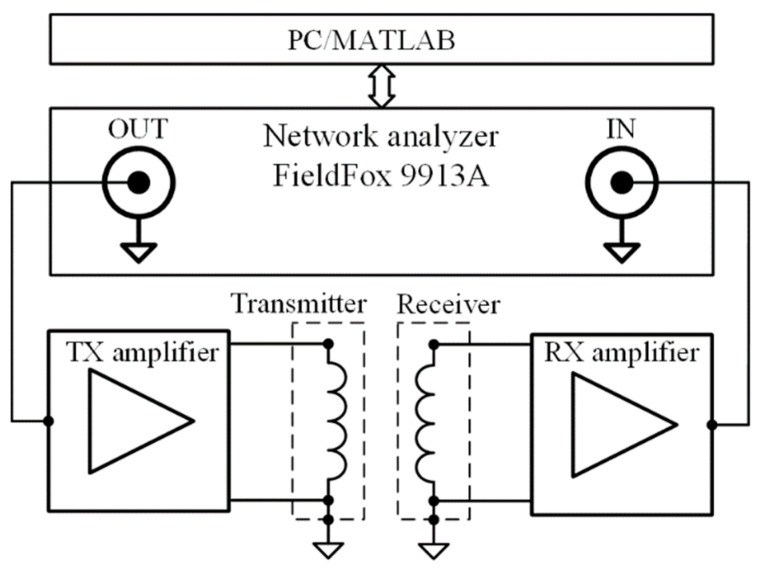
Block diagram of the experimental setup.

The experimental setup consisted of shielded coils in a transmitter-receiver sensor configuration, associated circuitry for signal amplification and filtering and network analyzer Keysight FieldFox 9913A. The electronics and network analyzer were battery-powered and isolated from the ground. The block diagram of the experimental setup is shown in Figure 1.

**Figure 2:**
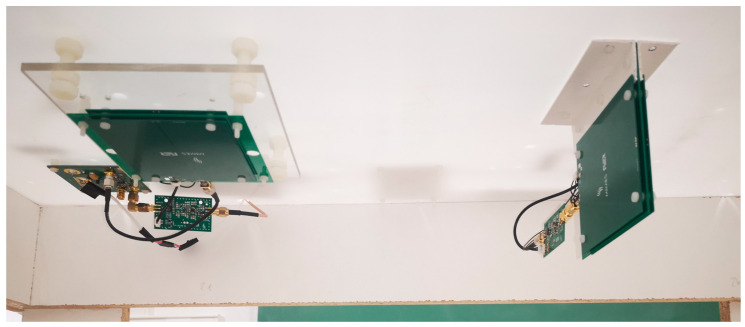
The photograph of the sensor: transmitter (**left**) and receiver (**right**).

**Figure 3:**
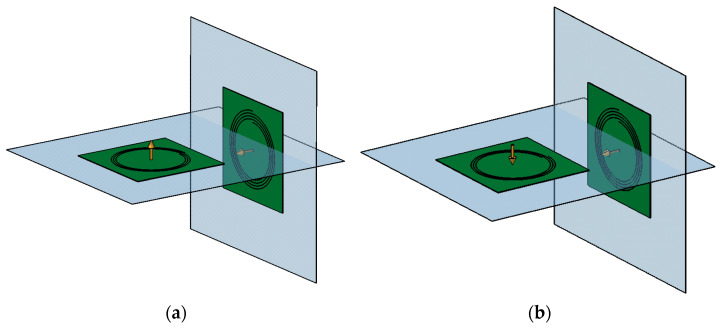
Sensor geometry: (**a**) horizontal transmitter coil oriented upwards, vertical receiver coil, (**b**) horizontal transmitter coil oriented downwards, vertical receiver coil.

The sensor and electronics were mounted on a stationary stand, and the intercoil separation could be adjusted to 27 cm or 37 cm before each measurement. A photograph of the sensor is shown in Figure 2. The transmitter and receiver coils were perpendicular to each other to reduce the direct inductive coupling, with the magnetic moment of the receiver coil directed horizontally towards the transmitter coil. The transmitter coil could be oriented so that its magnetic moment pointed either up or down, as shown in Figure 3. 

**Figure 4:**
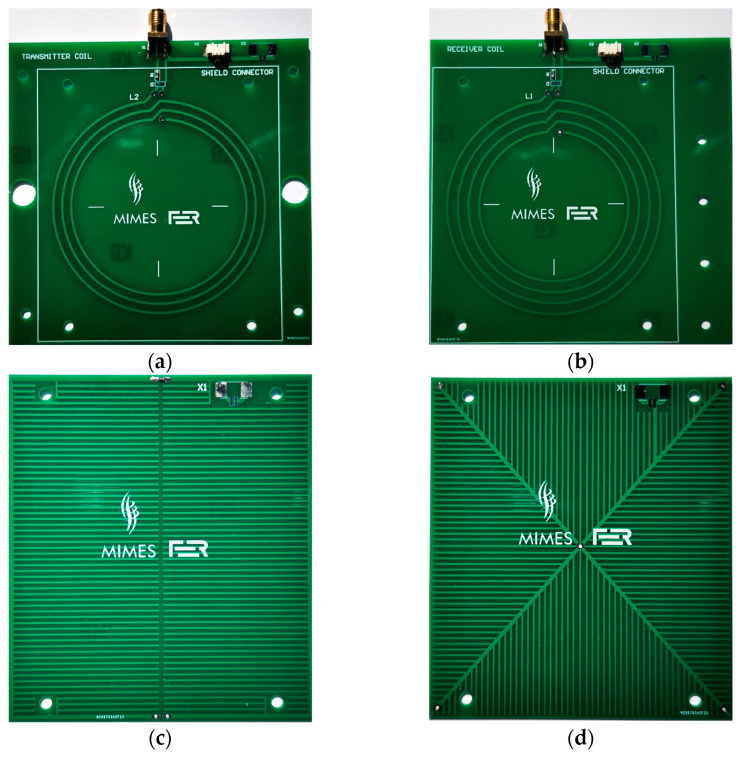
PCB coils and shields: (**a**) transmitter coil; (**b**) receiver coil; (**c**) comb C pattern shield; (**d**) comb X pattern shield.

The transmitter and receiver consisted of printed circuit board (PCB) coils and a PCB shield above and below each coil. FR-4 substrate for PCB was used with a thickness of 0.8 mm to reduce coil self-capacitance. The transmitter coil had 3 turns, and the receiver coil had 4 turns, as shown in Figure 4a,b. The outer radius of both coils was 35 mm, with an interturn spacing of 3.5 mm and a copper trace width of 0.8 mm. A slight height misalignment was introduced when changing the transmitter orientation due to the PCB thickness of 0.8 mm. The transmitter coil was vertically offset by 2.08 cm above the center of the receiver coil when oriented up and 2 cm when oriented down. Two different shield comb patterns were used: C pattern and X pattern, as shown in Figure 4c,d. Comb shielding patterns were used because their traces do not form inductive loops and they minimize conductive surface; thus, reducing the eddy current losses. Shield dimensions were 87.5 mm × 77.5 mm, copper trace width was 0.5 mm and the spacing between traces was 1.5 mm. Depending on the configuration, the spacing between shield and coil could be set to 2 mm, 4 mm or 6 mm. Shields were connected to the same reference potential as the coils.

The excitation signal from the network analyzer was fed into the transmitter amplifier, which consisted of a 4th order low-pass filter with a cutoff frequency of 35 MHz and the power amplifier with a gain of 8 dB. The amplifier and the transmitter were connected by a 20 cm coaxial cable. The receiver was connected to a 2nd order low pass filter with a cutoff frequency of 46 MHz to eliminate the coil ringing and ensure the amplifier stability. The receiver amplifier with a gain of 48 dB and a cutoff frequency of 35 MHz was connected to the receiver only with SMA connectors to avoid additional cable capacitance that would lower the resonant frequency of the coil.

A sensor transfer function was measured using the network analyzer Keysight FieldFox 9913A in 1001 logarithmically spaced frequencies from 1 MHz to 35 MHz. Additionally, impedance parameters of the coils, cables and filters required for sensor modelling were determined using the impedance analyzer Agilent 4294A.

### 2.2. Equivalent Circuit Model

**Figure 5:**
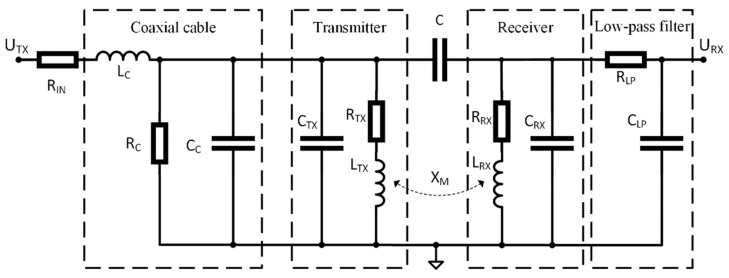
Equivalent circuit model of the experimental setup.

The equivalent circuit model of the experimental setup is shown in Figure 5. The transmitter and receiver parameters depend on the sensor configuration, whereas the coaxial cable and low-pass filter parameters remain unchanged.

The modelled transfer function *H* follows from the equivalent circuit model:

(1) $$H\left(\omega ,C,M,k\right)=\frac{U_{RX}}{U_{TX}},$$

where ω is the angular frequency, *C* is the mutual capacitance, *X_M_* = *jωM − kω*^2^ is the cross-impedance, *M* is the mutual inductance and factor *k* approximates frequency-dependent losses. Since the coupling is weak due to the transmitter-receiver separation and orientation, parameters *C*, *M* and *k* have low values compared to the other circuit parameters. Consequently, the transfer function *H* can be approximated using first-order Taylor expansion around (*C*, *M*, *k*) = (0, 0, 0):

(2) $$H\left(\omega ,C,M,k\right)\approx h_{C}\left(\omega \right)C+h_{M}\left(\omega \right)M+h_{k}\left(\omega \right)k,$$

where *h_C_*, *h_M_* and *h_k_* are partial derivatives of *H* with respect to *C*, *M* and *k*. This simplification is justified because the computations using typical values of the parameters from Section 3 show no observable difference between the exact expression for *H* and its approximation given in (2). Relevant expressions that constitute (2) are:

(3) $$Z_{IN}=R_{IN}+i\omega L_{c},$$

(4) $$Z_{TX}=R_{TX}+i\omega L_{TX},$$

(5) $$Z_{RX}=R_{RX}+i\omega L_{RX},$$

(6) $$Y=R_{C}^{-1}+Z_{IN}^{-1}+i\omega \left(C_{c}+C_{TX}\right),$$

(7) $$Z_{D}=Z_{IN}\left(1+YZ_{TX}\right)\left(1+i\omega C_{LP}R_{LP}+Z_{RX}\left(i\omega C_{RX}+i\omega C_{LP}-\omega^{2}C_{RX}C_{LP}R_{LP}\right)\right),$$

(8) $$h_{M}\left(\omega \right)=\frac{i\omega }{Z_{D}},$$

(9) $$h_{k}\left(\omega \right)=\frac{-\omega^{2}}{Z_{D}},$$

(10) $$h_{C}\left(\omega \right)=\frac{i\omega Z_{TX}Z_{RX}}{Z_{D}}.$$

Equations (4) and (5) are the transmitter and receiver impedances, *Z_TX_* and *Z_RX_*, without their capacitances *C_TX_* and *C_RX_*. There is a phase difference between *h_M_* in (8) and *h_C_* in (10) caused by the product of *Z_TX_* and *Z_RX_*_._ These impedances are dominantly inductive at frequencies above 1 MHz, with the phase close to 90°, which means that the phase difference between *h_M_* and *h_C_* is 180°. This is independent of the transmitter-receiver separation since only *C*, *M* and *k* depend on the separation.

### 2.3. Experimental Procedure and Model Parameter Estimation

The measured transfer function of the sensor is denoted as *H*_e_ and it was measured for the two orientations of the transmitter coil, as shown in Figure 3. First, the sensor transfer function *H*_e,up_ was measured with the transmitter oriented upwards, as shown in Figure 3a. Then, the transmitter coil was flipped downwards, and the transfer function *H*_e,down_ was measured, as shown in Figure 3b. The capacitive coupling was the same in both cases because the relative positions of all conductive surfaces (copper traces and parts of the experimental setup) were unchanged, while the inductive coupling changed sign and was more sensitive to the transmitter height misalignment between the two orientations.

The equivalent circuit parameters needed to calculate *h_C_*, *h_M_* and *h_k_* were determined with the impedance analyzer before measuring the sensor transfer function. The values of *C, M* and *k* were estimated by fitting (2) to the measured transfer functions *H*_e,up_ and *H*_e,down_ using linear least-squares regression over the frequency range of interest. For each measured frequency ω, the equation pair was:

(11) $$H_{e,\mathrm{up}}(\omega )=h_{C}(\omega )\cdot C+h_{M}(\omega )\cdot M+h_{k}(\omega )\cdot k,$$

(12) $$H_{e,\mathrm{down}}(\omega )=h_{C}(\omega )\cdot C-h_{M}(\omega )\cdot M-h_{k}(\omega )\cdot k.$$

In the case of *H*_e,up_, the contributions of the inductive coupling parameters *M* and *k* had a positive sign and negative in the case of *H*_e,down_. The overdetermined system with *2n* equations, where *n* was the number of measurement frequencies, was then:

(13) $$\left[\begin{array}{c} H_{e,\mathrm{up}}(\omega_{1})\\ H_{e,\mathrm{down}}(\omega_{1})\\ \vdots \\ \vdots \\ H_{e,\mathrm{up}}(\omega_{n})\\ H_{e,\mathrm{down}}(\omega_{n}) \end{array}\right]=\left[\begin{array}{ccc} h_{C}(\omega_{1}) & h_{M}(\omega_{1}) & h_{k}(\omega_{1})\\ h_{C}(\omega_{1}) & -h_{M}(\omega_{1}) & -h_{k}(\omega_{1})\\ & \vdots & \\ & \vdots & \\ h_{C}(\omega_{n}) & h_{M}(\omega_{n}) & h_{k}(\omega_{n})\\ h_{C}(\omega_{n}) & -h_{M}(\omega_{n}) & -h_{k}(\omega_{n}) \end{array}\right]\left[\begin{array}{c} C\\ M\\ k \end{array}\right].$$

Since the phase difference between *h_C_* and *h_M_* is 180°, capacitive and inductive coupling components $h_{C}(\omega )\cdot C$ and $h_{M}(\omega )\cdot M$ in (11) have opposite phases, which reduces the overall coupling. In the case of (12), $h_{M}(\omega )\cdot M$ changes the sign, so the capacitive and inductive coupling components are now in phase, and the overall coupling is increased.

### 2.4. Summary of the Method for the Electrostatic Shielding Evaluation

The above procedure is applicable to EMI sensors that are weakly coupled, i.e., where the transmitter and receiver coils are separated such that the mutual inductances and capacitances are several orders of magnitude smaller than the values of the individual coil inductances and capacitances. This includes, for example, EMI soil sensors and tomography and spectroscopy systems. The procedure can be summarized as a method for evaluation of the electrostatic shielding effectiveness as follows:

1. Derive the equivalent circuit model of an HFEMI sensor for the case where the medium under study is not present, i.e., the sensor in the air.
2. Linearize the derived model with respect to the inductive and capacitive coupling parameters.
3. Measure the impedance for each shielded coil of the sensor and estimate the parameters of its equivalent circuit using the laboratory impedance analyzer. The estimated parameters are used as the parameters of the HFEMI sensor linearized model.
4. Measure the sensor responses for two opposing transmitter orientations. Alternatively, if the transmitter cannot be reoriented, change the excitation current direction.
5. Estimate the coupling parameters from the linearized model and the measured sensor response.

## 3. Results and Discussion

### 3.1. Measurement of Shielded Transmitter and Receiver Equivalent Circuit Parameters

**Figure 6:**
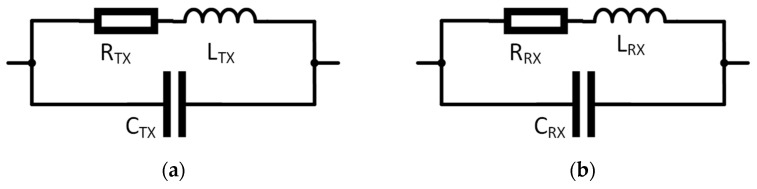
Equivalent circuit parameters model: (**a**) transmitter and (**b**) receiver.

Transmitter and receiver equivalent circuit model parameters, shown in Figure 6, for each coil and shield combination are given in Table 1, as provided by the Agilent 4294A impedance analyzer. The equivalent circuit parameters for the coaxial cable and the low-pass filter are given in Table 2. The filter capacitance *C_LP_* is the sum of the receiver amplifier input capacitance and the filter capacitor.

The columns in Table 1, Table 3 and Table 4 represent different shield patterns (C or X) and coil-shield spacing in millimeters, e.g., C-4 denotes a C pattern shield at 4 mm spacing from a coil. Receiver capacitance *C_RX_* and transmitter capacitance *C_TX_* include coil self-capacitance and coil-shield capacitance that depend on the shielding pattern and spacing from the coil. Receiver inductance *L_RX_*, transmitter inductance *L_TX_* and their respective series resistances *R_RX_* and *R_TX_* are defined by the coil geometry and the skin and proximity effects in the copper traces of the coils and shields. 

### 3.2. Estimation of Coupling Parameters

The coupling parameters *C*, *M* and *k* were estimated using the procedure in Section 2.3 and the measurement data up to 20 MHz to avoid the coil resonance effects. The results are given in Table 3 for the transmitter-receiver separation of 27 cm and in Table 4 for the 37 cm separation. A total of 36 different sensor configurations were tested (6 × 6, two shield patterns and three coil–shield spacings for the transmitter and for the receiver). Two measurements were made for each configuration: first with the transmitter coil oriented upwards, and then oriented downwards.

For a given transmitter-receiver separation, the mutual inductance *M* and losses *k* were similar for all configurations, which meant that the inductive coupling was not significantly affected by changing the shield pattern and coil–shield spacing. The mutual capacitance *C* depended on the configuration. It could be negligible in some configurations (estimated *C* was zero), but generally increased as the coil–shield spacing increased.

### 3.3. Effects of Shielding on the Sensor Transfer Function

**Figure 7:**
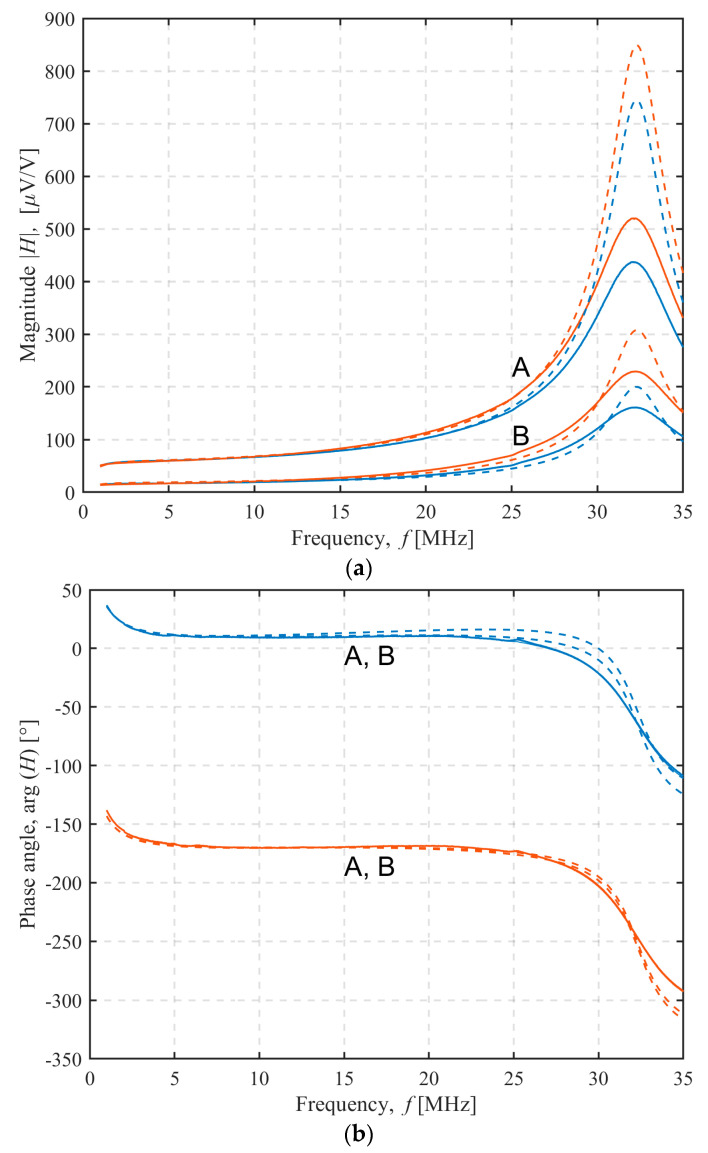
TX C-6/RX C-6 configuration: (**a**) magnitude and (**b**) phase characteristics of the sensor. Measurement (full line) and model results (dashed lined). Transmitter orientation up (blue line) or down (red line). Transmitter–receiver separation 27 cm (**A**) or 37 cm (**B**).

The magnitude and phase spectra of the sensor transfer function *H* for TX C-6/RX C-6 sensor configuration are shown in Figure 7 in the frequency range from 1 MHz to 35 MHz, depending on the transmitter orientation (blue for up, red for down) and transmitter-receiver separation (A for 27 cm, B for 37 cm). The resonant peak for all these cases in Figure 7 was at 32 MHz, and this was primarily determined by the receiver parameters (*L_RX_* and *C_RX_*) and the input impedance of the low-pass filter and receiver amplifier.

In the examples shown, the sensor transfer functions for a given separation were similar for both transmitter orientations of up to 13 MHz, after which the difference became more pronounced. This was due to the inductive and capacitive components of the coupling, which, according to the model, were mutually either in phase or 180° out of phase. When the transmitter was oriented downwards, the inductive and capacitive coupling components were in phase, and the receiver voltage increased. By orienting the transmitter upwards, the phase angle of the inductive coupling flipped, whereas the capacitive coupling remained unchanged, so the receiver voltage was now reduced.

In addition to being dependent on the transmitter orientation, the sensor transfer function magnitude for the 27 cm separation was approximately 3.5 times larger than for the 37 cm separation. However, the phase of the receiver voltage did not depend noticeably on the separation, as shown in Figure 7b, indicating that the phase relationship between the inductive and capacitive components remained unchanged for the response in the air. This observation was also backed by the model.

The dashed lines in Figure 7 show the magnitude and phase spectra calculated from the model using the electromagnetic coupling parameters from Table 3 and Table 4. The model results and the measurements were in agreement up to 20 MHz. Since the model did not take into account the resonance damping of the copper traces skin effect and coil–shield proximity effect, the upper frequency limit of the further experimental analysis was set at 20 MHz.

**Figure 8:**
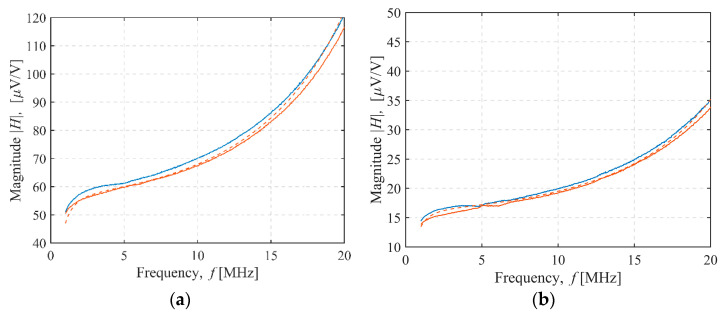
TX C-2/RX C-2 configuration sensor transfer function magnitude. Transmitter–receiver separation: (**a**) 27 cm, (**b**) 37 cm. Measurement (full line) and model results (dashed line). Transmitter orientation up (blue line) or down (red line).

**Figure 9:**
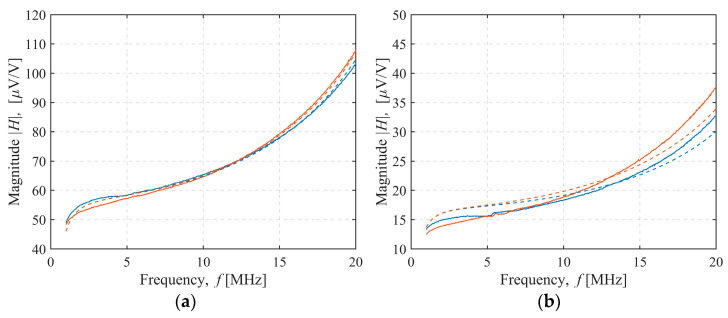
TX C-4/RX X-4 configuration sensor transfer function magnitude. Transmitter–receiver separation: (**a**) 27 cm, (**b**) 37 cm. Measurement (full line) and model results (dashed line). Transmitter orientation up (blue line) or down (red line).

**Figure 10:**
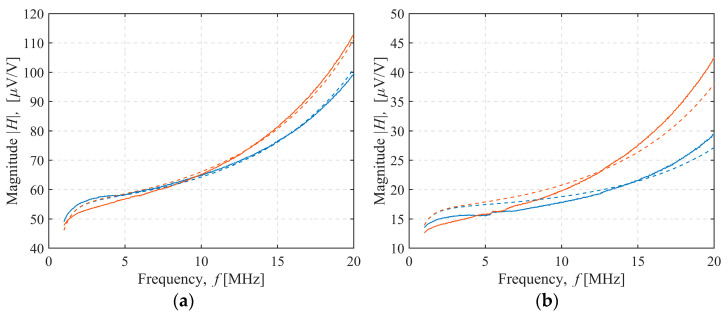
TX X-4/RX X-4 configuration sensor transfer function magnitude. Transmitter–receiver separation: (**a**) 27 cm, (**b**) 37 cm. Measurement (full line) and model results (dashed line). Transmitter orientation up (blue line) or down (red line).

**Figure 11:**
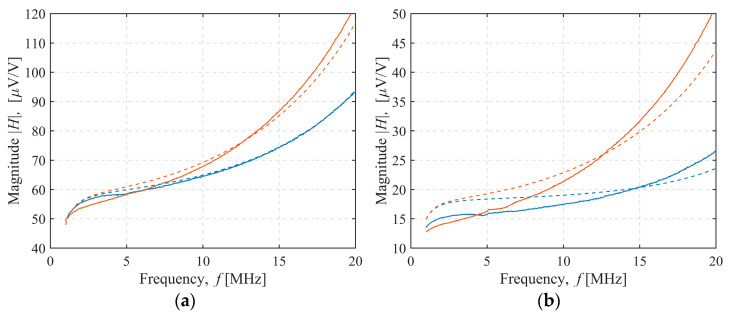
TX X-6/RX X-6 configuration sensor transfer function magnitude. Transmitter–receiver separation: (**a**) 27 cm, (**b**) 37 cm. Measurement (full line) and model results (dashed line). Transmitter orientation up (blue line) or down (red line).

The magnitude of the measured and modeled sensor transfer function for 4 of the 36 sensor configurations tested was shown in Figure 8, Figure 9, Figure 10 and Figure 11. In Figure 8, both the transmitter and receiver had C-shields spaced 2 mm apart (TX C-2/RX C-2 configuration). There was only a slight, frequency-independent difference between the spectra for both transmitter orientations, which was due to small changes in mutual inductance that occurred when the transmitter orientation was changed manually, resulting in a height misalignment between the transmitter orientations. No other differences were observed, and the estimated mutual capacitance *C* was zero, as shown in Table 3 and Table 4, resulting in identical modeled transfer functions for both orientations. In Figure 9, the transmitter had a C-shield, and the receiver had an X-shield, both spaced 4 mm apart (TX C-4/RX X-4), as shown in Figure 10, and both had X-shields spaced 4 mm apart (TX X-4/RX X-4), while in Figure 11, the X-shields were spaced 6 mm apart (TX X-6/RX X-6). In contrast to Figure 8, the differences between the magnitudes of the sensor transfer functions for both transmitter orientations in all these configurations clearly increased with the frequency and the coupling capacitance *C*. The lowest coupling capacitance *C* was found in Figure 9, and the highest in Figure 11, Table 3 and Table 4. The capacitive coupling *C* was lower for higher capacitances *C_RX_* and *C_TX_*, as shown in Table 1. Capacitances *C_RX_* and *C_TX_* were higher at smaller spacings and slightly higher with C-shields than with X-shields, as shown in Table 1. At the lower frequencies, the difference between the magnitude spectra for both transmitter orientations was dominantly caused by the small changes in mutual inductance due to the transmitter height misalignment, but this was overcome by the capacitive coupling that became a dominant cause of the difference at the crossing frequency near 5 MHz.

### 3.4. Shield Effectiveness Validation in the Presence of Conductive Medium

**Figure 12:**
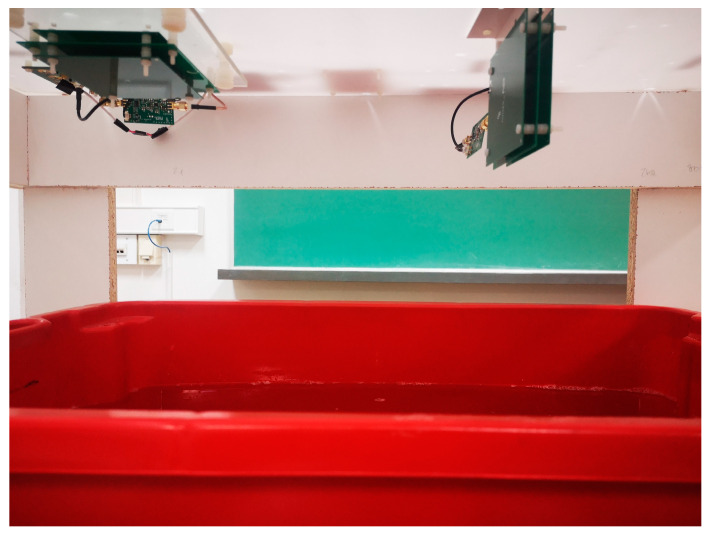
The photograph of the sensor above the container filled with saline water.

To demonstrate the effect of shielding, the sensor response was measured above a container filled with 60 L of saline water with an electrical conductivity of 1 S/m and a dielectric permittivity of 78, as shown in Figure 12. The saline water was used to emulate the soil because it has similar electromagnetic properties, and allowed for easier control of the electrical conductivity. First, the response of the sensor in the air was measured and then the response above the saline water was measured. Two different shield configurations were evaluated (TX C-2/RX C-2 and TX X-6/RX X-6). The intercoil separation was 27 cm and the sensor lift-off above the saline water was 11 cm with respect to the transmitter. The experimental setup and instrumentation were the same as in Section 2.1.

**Figure 13:**
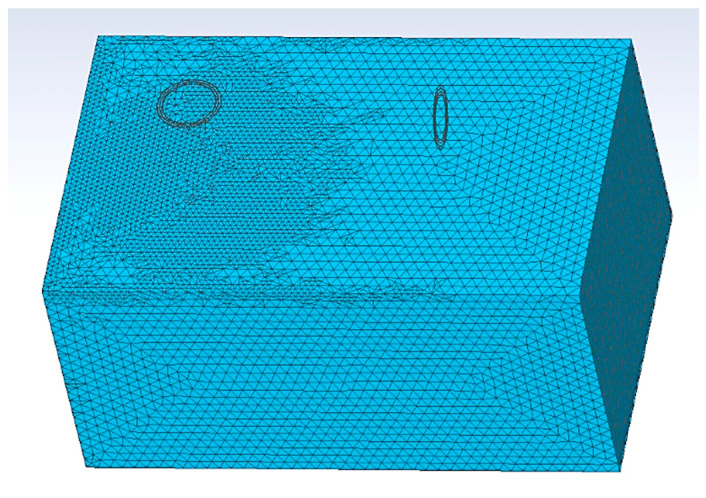
Sensor geometry and mesh grid of the medium used in FEM analysis.

**Figure 14:**
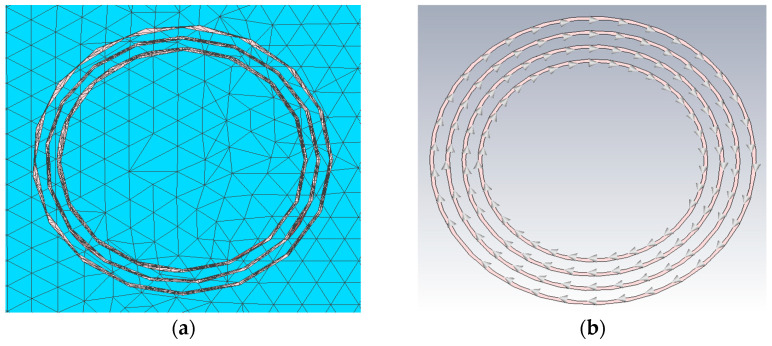
Coil design used in FEM analysis: (**a**) transmitter coil; (**b**) receiver coil.

The measurement results were compared with the 3D full-wave finite element method (FEM) analysis using the CST studio suite 2021 software (Dassault Systèmes Simulia) [23]. The FEM model, equivalent to the measurement setup geometry, is shown in Figure 13. The transmitter and receiver coils, shown in Figure 14, were modeled as a superposition of individual coil loops. The saline water was modeled as a homogeneous medium with dimensions of 58.3cm × 37.2cm × 28.3 cm, the electrical conductivity of 1 S/m and dielectric permittivity of 78. To simulate the sensor response in air, the medium parameters were set to an electric conductivity of 0 S/m and dielectric permittivity of 1. The boundary of the FEM model was set at 100 cm from the medium. The mesh grid consisted of 580,133 elements.

The sensor transfer function for the response in the air is *H_air_*, and the receiver voltage is the primary induced voltage *U_P_* due to the direct coupling. The transfer function above the conductive medium is *H_medium_*, and the receiver voltage is the sum of the primary voltage *U_P_* and the secondary voltage due to the coupling through the medium *U_S_*. A standard representation of the measurement results in the EMI soil sensing is the ratio of secondary to primary induced voltage, which cancels the instrumentation and excitation related terms and allows the comparison with numerical models [5].

(14) $$H_{air}=\frac{U_{P}}{U_{TX}},$$

(15) $$H_{medium}=\frac{U_{P}+U_{S}}{U_{TX}},$$

(16) $$\frac{U_{S}}{U_{P}}=\frac{H_{medium}-H_{air}}{H_{air}}.$$

**Figure 15:**
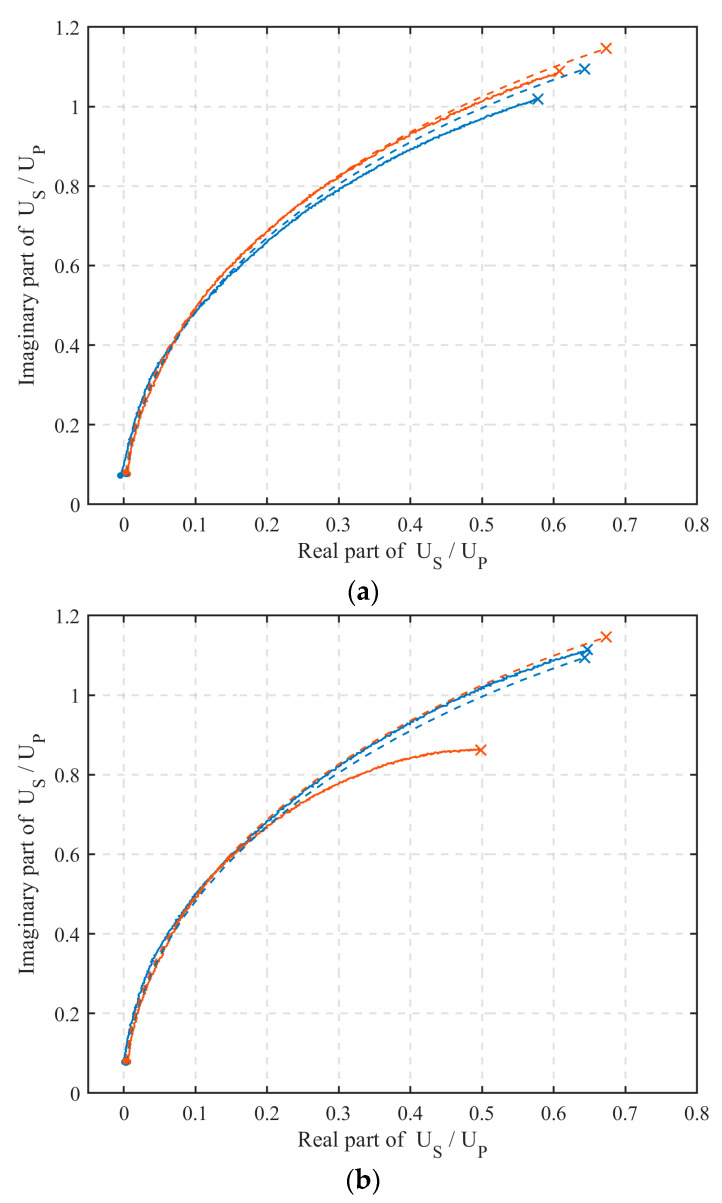
Ratio of secondary and primary induced voltage: (**a**) TX C-2/RX C-2 configuration, (**b**) TX X-6/RX X-6 configuration. Measurement (full line) and FEM analysis (dashed lined). Transmitter orientation up (blue line) or down (red line).

The measurement results for the two sensor configurations (TX C-2/RX C-2 and TX X-6/RX X-6) and the FEM results are shown in the complex plane in Figure 15. The measurements (full line) were compared with the FEM analysis (dashed line) for both transmitter orientations (blue line for up and red line for down) in the frequency range from 1 MHz (dot) to 20 MHz (cross). An effective shield reduced the capacitive coupling component of both the primary and secondary voltages, so that their ratio was predominantly due to inductive coupling and, consequently, was in better overall agreement with the FEM model results. In Section 2, it was shown that the TX C-2/RX C-2 configuration had negligible capacitive coupling compared to the TX X-6/RX X-6 configuration; hence, a better overall agreement of the former with the FEM model. This case study demonstrated that the method in Section 2 was useful in evaluating the effectiveness of a shield.

## 4. Conclusions

This study presented a method to evaluate the shielding effectiveness in electromagnetic induction transmitter-receiver sensor configurations. The measured sensor transfer characteristics in the air from 1 MHz to 20 MHz for two transmitter coil orientations could be explained using the presented model that additively included the capacitive coupling, the inductive coupling and the losses. The model could be used to estimate the coupling capacitance and inductance from the measurements and these could be used to evaluate the effectiveness of the shielding. We illustrated this on 36 different shielding configurations. The results were in accordance with the established shielding practices as they showed that the lowest capacitive coupling was achieved primarily with lower coil–shield spacing and, to a lesser extent, with the choice of the shield pattern. The comparison of the FEM results and the measurements with two shield configurations above the saline solution confirmed that the measurement with the shield that had a negligible estimated capacitive coupling yielded a better overall agreement.

## Figures and Tables

**Table 1:** Transmitter and receiver equivalent circuit parameters.

	C-2	C-4	C-6	X-2	X-4	X-6
***L_TX_* (μH)**	1.101	1.117	1.124	1.084	1.108	1.117
***C_TX_* (pF)**	12.05	9.78	9.04	11.68	9.58	8.77
***R_TX_* (Ω)**	0.345	0.351	0.347	0.301	0.29	0.286
***L_RX_* (μH)**	1.489	1.517	1.528	1.485	1.519	1.527
***C_RX_* (pF)**	13.71	10.73	9.47	13.27	10.53	9.24
***R_RX_* (Ω)**	0.399	0.386	0.404	0.428	0.421	0.409

**Table 2:** Coaxial cable and low-pass filter equivalent circuit parameters.

	Coaxial Cable
***L_k_* (nH)**	40
***C_C_* (pF)**	18.5
***R_k_* (kΩ)**	716
	**Low-pass filter**
***C_LP_* (pF)**	6.9
***R_LP_* (Ω)**	499

**Table 3:** Model parameters, 27 cm intercoil separation.

		Transmitter Coil (TX)						
		C-2	C-4	C-6	X-2	X-4	X-6	
**Receiver coil (RX)**	**C-2**	66.63	68.11	67.59	65.67	66.85	67.66	*M* (pH)
	**C-4**	68.06	68.58	68.24	66.92	67.87	65.25	
	**C-6**	69.22	69.71	69.58	68.48	69.31	69.68	
	**X-2**	62.69	63.83	63.55	61.34	62.68	63.09	
	**X-4**	65.31	66.02	65.13	64.16	65.5	64.89	
	**X-6**	67.83	68.63	68.36	67.24	68.29	68.49	
	**C-2**	5.1	5.2	5.2	4.9	5	5	*k* (0.1 pH/MHz)
	**C-4**	5.2	5.3	5.1	5	5.1	5	
	**C-6**	5.3	5.4	5.2	5.3	5.4	5.4	
	**X-2**	4.8	4.8	4.9	4.6	4.8	4.8	
	**X-4**	4.9	5	5.1	5	5.1	5	
	**X-6**	5.3	5.3	5.6	5.8	5.4	5.4	
	**C2**	0	0	0	0	0	0	*C* (10^−17^ F)
	**C4**	0	0	3.6	1.3	2.8	8.7	
	**C6**	0	2.7	10.3	8.1	14.2	21.2	
	**X2**	0	0	3.3	0	2.9	9.8	
	**X4**	0	3.2	10.4	9.8	14.4	23.2	
	**X6**	0	8	18.2	16.4	24.3	34.7	

**Table 4:** Model parameters, 37 cm intercoil separation.

		Transmitter Coil (TX)						
		C-2	C-4	C-6	X-2	X-4	X-6	
**Receiver coil (RX)**	**C-2**	19.09	19.39	18.91	18.76	19.23	18.7	*M* (pH)
	**C-4**	20.02	20.23	20.59	19.75	20.27	20.34	
	**C-6**	21.12	21.88	21.32	20.79	21.85	21.3	
	**X-2**	18.03	18.23	19.31	17.82	18.2	19.25	
	**X-4**	19.56	19.68	20.21	19.45	19.82	20.19	
	**X-6**	21.07	21.26	21.22	20.88	21.44	21.31	
	**C-2**	0.7	0.7	0.7	0.7	0.7	0.7	*k* (0.1 pH/MHz)
	**C-4**	0.8	0.8	0.8	0.8	0.8	0.8	
	**C-6**	0.9	0.9	0.8	0.9	0.9	0.9	
	**X-2**	0.7	0.7	0.8	0.7	0.7	0.8	
	**X-4**	0.8	0.8	0.8	0.8	0.8	0.8	
	**X-6**	0.9	0.9	0.9	0.9	0.9	0.9	
	**C2**	0	0	0	0	0	0.6	*C* (10^−17^ F)
	**C4**	0	1.5	5.7	4.7	6.2	10.3	
	**C6**	0.5	5.3	11	10.3	15.3	20.2	
	**X2**	0	2.2	5.4	5	8.4	11	
	**X4**	1.1	5.6	11.4	10.8	15.8	20.5	
	**X6**	2.6	9.7	17.8	16.8	24.6	31	

## Data Availability

The data presented in this study are available on request from the corresponding author.
